# From E-Commerce to the Metaverse: A Neuroscientific Analysis of Digital Consumer Behavior

**DOI:** 10.3390/bs14070596

**Published:** 2024-07-13

**Authors:** Alessandro Fici, Marco Bilucaglia, Chiara Casiraghi, Cristina Rossi, Simone Chiarelli, Martina Columbano, Valeria Micheletto, Margherita Zito, Vincenzo Russo

**Affiliations:** 1 Department of Business, Law, Economics and Consumer Behaviour “Carlo A. Ricciardi”, Università IULM, 20143 Milan, Italy; alessandro.fici@iulm.it (A.F.); marco.bilucaglia@studenti.iulm.it (M.B.); cristina.rossi26@studenti.iulm.it (C.R.); simone.chiarelli@studenti.iulm.it (S.C.); martina.columbano@studenti.iulm.it (M.C.); valeria.micheletto@iulm.it (V.M.); margherita.zito@iulm.it (M.Z.); vincenzo.russo@iulm.it (V.R.); 2 Behavior and Brain Lab IULM—Neuromarketing Research Center, Università IULM, 20143 Milan, Italy

**Keywords:** metaverse, Second Life, digital consumer behavior, consumer neuroscience, neuromarketing, shopping experience, EEG, emotions

## Abstract

The growing interest in consumer behavior in the digital environment is leading scholars and companies to focus on consumer behavior and choices on digital platforms, such as the metaverse. On this immersive digital shopping platform, consumer neuroscience provides an optimal opportunity to explore consumers’ emotions and cognitions. In this study, neuroscience techniques (EEG, SC, BVP) were used to compare emotional and cognitive aspects of shopping between metaverse and traditional e-commerce platforms. Participants were asked to purchase the same product once on a metaverse platform (Second Life, SL) and once via an e-commerce website (EC). After each task, questionnaires were administered to measure perceived enjoyment, informativeness, ease of use, cognitive effort, and flow. Statistical analyses were conducted to examine differences between SL and EC at the neurophysiological and self-report levels, as well as between different stages of the purchase process. The results show that SL elicits greater cognitive engagement than EC, but it is also more mentally demanding, with a higher workload and more memorization, and fails to elicit a strong positive emotional response, leading to a poorer shopping experience. These findings provide insights not only for digital-related consumer research but also for companies to improve their metaverse shopping experience. Before investing in the platform or creating a digital retail space, companies should thoroughly analyze it, focusing on how to enhance users’ cognition and emotions, ultimately promoting a better consumer experience. Despite its limitations, this pilot study sheds light on the emotional and cognitive aspects of metaverse shopping and suggests potential for further research with a consumer neuroscience approach in the metaverse field.

## 1. Introduction

As the world undergoes a constant process of digitization in which experiences become more immersive and smart technologies saturate the market, consumer behavior continues to evolve and adapt to new and engaging environments [1], urging a new understanding of how these realities impact consumer needs, perceptions, desires, motivations, insights, and ultimately practices [2].

### 1.1. The Metaverse as a New Frontier for Digital Commerce

One of the most effective and immersive ways for consumers to interact in the digital space is the metaverse [3], a multiuser environment that transcends traditional boundaries and merges physical reality with digital virtuality [4]. Interest in the metaverse is growing rapidly, with an estimated financial investment of 800 billion dollars in 2024 [5] and an extensive list of possible applications [6]. These include education, teaching and learning [7,8,9], hospitality and tourism [10,11], virtual job creation [12], healthcare and medicine [13,14], education [15,16], games and leisure activities [17,18], digital cosmetics try-on [19] and digital shopping experiences [20].

The sense of presence through the use of avatars and the occurrence of constant immersive interactions (from both experiential and economic points of view) with identities and objects [21] have led retailers and academics to recognize the central role of the metaverse in reshaping the marketing and shopping experience [22,23]. On metaverse platforms, consumers’ avatars can not only engage with brands and purchase physical or intangible goods but also explore digital stores, test products, and interact with avatars of salespeople and other customers [24]. If traditional shopping has set the stage for e-commerce, the metaverse offers a distinctive digital shopping experience [25] that recreates the atmosphere of a brick-and-mortar store in digital retail [26] and offers promising possibilities such as three-dimensional product visualization [27]. This explains why the metaverse is attracting major retail brands such as Adidas, Gucci, Balenciaga, Nike, Louis Vuitton, Burberry, Tommy Hilfiger, Dolce & Gabbana, and Ralph Lauren as well as fast fashion retailers such as H&M, Zara, and Forever 21 [28]. 

The metaverse is considered the evolution of e-commerce [25,29], even if it faces many challenges, from established, highly accessible online shopping platforms [23] to socio-cultural barriers, such as the digital divide [30]. In addition, cognitive and mental effort [31] and an overall negative emotional experience [32] could limit acceptance. Therefore, it is important to investigate the actual role of metaverse platforms in digital consumer behavior as companies and users invest in them [33,34], as well as their objective limitations [6], especially regarding first-generation platforms such as Second Life (SL) [35]. In this paper, we focus on one of the first and most popular metaverse platforms that is still heavily used today: SL.

### 1.2. Second Life as a Part of the Metaverse

SL is an immersive, highly active virtual world [36,37] in which many companies are continuously investing due to its more than 70 million registered accounts and monthly growth of around 350,000 new accounts [34]. Unlike other popular metaverse platforms such as Roblox and Minecraft, which are mainly categorized as online gaming platforms [38] and have few photorealistic graphics, SL is not a video game [39]. In addition, this platform is characterized by a marketplace and extensive possibilities for buying and selling [40], which has led to the development of a thriving digital economy within SL [40]. This makes SL a metaverse platform suitable for exploring digital consumer behavior in the metaverse. The extensive use of SL over the years has been favored by the high degree of personalization [41] and the immersive nature of the environment, which is reportedly more enjoyable and socially interactive and can better encourage buying and selling processes than a real-world retail environment or e-commerce [42,43]. In the SL marketplace, purchases are made by avatars who exchange real currency for the platform’s virtual currency, Linden Dollars (L$) [44]. SL not only serves as a digital business environment, but also offers several benefits, including the opportunity to earn real money and create interactive experiences that captivate users [44]. In fact, several real-world companies have chosen to open virtual stores in SL [33], including Adidas, American Apparel, Dell, Harvard Law School, IBM, Microsoft, Pontiac, Sony Ericsson, and Toyota [45]. Although SL is commonly regarded as a first-generation platform [6], the SL marketplace is still very active and productive in terms of assets and active users [33,34], with the authors comparing it to traditional e-commerce to obtain data on trade interactions [46] and normative issues [33]. Therefore, SL continues to play a crucial role in the metaverse [6,47] as a new frontier for digital commerce with marketing activities targeting younger generations [48]. As SL is a fully fledged active platform for digital shopping experiences [6,35], its activity has consolidated over time [33,34], so much so that several authors have highlighted its immersive and personalized experience that can surpass traditional e-commerce [43]. Therefore, instead of focusing on exploring the engagement and enjoyment of the shopping experience solely through traditional methods [49,50], it might be useful to examine it through a neuroscience lens as well. This approach could lead to a better understanding of the shopping experience in the metaverse [51,52] and promote a better understanding of its role compared to traditional e-commerce. This would also be useful in providing feedback to companies for their current and future investments in SL based on the physiological reactions of the subjects. 

### 1.3. The Role of Consumer Neuroscience in Studying the Metaverse

The use of neuroscience to analyze experiences in the metaverse is considered of great importance [52] and is supported by the need to deeply understand the role of digital environments in our cognitive and emotional processes [53]. 

However, although some attempts have been made to understand the role of consumer emotions and cognition in the metaverse [32,54,55], there is still limited scientific research on this topic [32,52]. This significant gap in the understanding of metaverse consumer experience [56] requires deeper exploration [52] to recognize how these aspects may differ between Second Life (SL) and traditional e-commerce. Understanding emotions, perceptions, and cognitive experiences in such a multisensory environment can explain consumer behavior [32] and the role of environmental features in modifying cognitive perceptions and emotions [57]. 

The use of neuroscientific techniques to study consumers’ brain processes is known as consumer neuroscience and, despite its emergence in the early 2000s, is currently on the rise [58]. Through the use of neuroscience tools such as electroencephalogram (EEG), eye tracking, skin conductance (SC), photoplethysmogram (PPG), electromyogram, and facial expressions, consumer neuroscience enables a deeper understanding of consumers’ emotions and cognitions in response to experiences in virtual spaces [59,60], websites [61], and mobile applications [62], as well as stimuli such as video advertisements [63,64,65,66] or product packaging [67,68,69]. In fact, measurements of consumer behavior and decision-making processes based on the registration of neurophysiological parameters can be more reliable and accurate, as they lack the mediation of cognitive processes [70,71]. 

The aforementioned work by Mandolfo and colleagues [32] examined consumer emotions in immersive environments such as augmented reality (AR), virtual reality (VR), and mixed reality (MR) using neuroscience techniques. This study highlights the importance of heightened arousal and emotional experience for an enhanced virtual experience and represents a first step towards integrating neuroscience techniques into metaverse-related emotion research. Recent studies on VR environments have utilized neuroscience techniques such as eye tracking [72] and EEG to assess cognitive load [56], affective states [32], and engagement [73], effectively demonstrating the essential contribution of neurophysiological measures to our understanding of emerging digital technologies and the way we interact with them. 

Among the studies that have investigated metaverse shopping through neuroscience, a study by Saffari and colleagues [74] found that frontal asymmetry in the gamma band changes over time and differs between planned and unplanned choices. Another study employed EEG, showing how a virtual educational environment can enhance students’ interactivity, immersion, cognition, and understanding accuracy [75]. However, as Costa-Feito and colleagues noted, neuroscience tools such as EEG are not widely used to analyze consumers’ emotions and perceptions on virtual platforms such as the metaverse [76]. These tools, combined with traditional surveys, could significantly improve our understanding of purchasing behavior in the metaverse compared to traditional e-commerce sites. To fill this gap in understanding the cognitive and emotional aspects of the metaverse consumer experience, a holistic approach that integrates traditional and neuroscientific tools is thus required.

In addition to consumer neuroscience, there are indeed comprehensive frameworks that analyze human behavior in dealing with new technologies. Davis’s Technology Acceptance Model (TAM) [77] provides a structured approach to assessing how consumers accept and use new technologies. Other variables that have been shown to have an impact on the digital consumer experience [78,79] include flow, i.e., how much a person feels cognitively efficient, motivated, and happy at the same time during an experience [80], and cognitive effort, i.e., how cognitively demanding the experience is [81]. By integrating consumer neuroscience tools with these metrics, it is possible to study the consumer experience in the metaverse in depth and gain deeper insights into consumer acceptance and interaction. 

### 1.4. Research Objectives

As consumers and industries continue to adopt novel digital environments such as the metaverse, there is a need to delve deeper into the consumer experience within this realm. In particular, our research will focus on exploring how these two digital shopping experiences may differ cognitively and emotionally. Focusing on the sustained popularity of SL and adopting a consumer neuroscience approach to explore cognitive and emotional variables in depth, our research aims to answer the following questions:

**RQ1:**  *From a neurophysiological perspective, does SL perform better than traditional e-commerce in terms of cognitive and emotional engagement?*

**RQ2:**  *Through the analysis of technology acceptance (TAM), optimal experience (flow), and cognitive effort, is the experience on SL perceived to be better than traditional e-commerce?*

Regardless of these research questions, a secondary goal of the article is to understand whether the neurophysiological indices used in this work are able to capture the emotional and cognitive experience on metaverse platforms, giving a contribution to future research on the topic. 

## 2. Materials and Methods

### 2.1. Sample

The study included a total of 33 subjects aged 20 to 31 years (M = 22.394, SD = 2.499), who thus belong to both Generation Z and Millennials or Generation Y [82], among the generations most engaged with the platforms of the metaverse [83]. The choice of sample size is in line with consumer neuroscience studies [84] and with several articles measuring neurophysiological parameters (e.g., [85,86,87]). A sensitivity analysis performed using G*Power [88] based on a repeated-measures model (total sample size of 33, 1 group, 8 measurements, α = 0.05, 1 − β = 0.95, ρ = 0.5, ϵ = 1) showed a minimum detectable effect size of f = 0.147, interpreted as “small” to “medium” [89]. This can be deemed adequate, considering the median effect size in cognitive neuroscience and experimental psychology of d = 0.93 [90], interpreted as more than “large” [89].

The sample was non-significantly gender-unbalanced in terms of proportions (binomial test: M = 54.5%, *p* = 0.728) and mean age (Mann–Whitney U = 21.5, *p* = 0.542). Only participants with at least a minimum level of knowledge of metaverse platforms were enrolled, to avoid potential discomfort that could compromise the authenticity of the experience. To prove this prerequisite level of knowledge, participants were first asked about their familiarity with platforms such as Roblox, Second Life, Minecraft, and Zepeto, and only those who demonstrated substantial rather than sporadic or occasional use were selected. 

The experiment was conducted according to the Declaration of Helsinki, and written consent was obtained from each participant. The consent form outlined the research objectives, the voluntary nature of the study participation, the guarantee of anonymity, and the non-invasive use of instrumentation. 

### 2.2. Instrumentation

The EEG signal was acquired through the NVX-52 device (Medical Computer Systems, Ltd., Moscow, Russia) from 38 Ag/AgCl electrodes evenly placed on the scalp (the exact locations can be found in [66]), 2 Ag/AgCl clips on the left and right earlobes and 1 Ag/AgCl adhesive patch on the right mastoid (M2). The montage was monopolar, referenced to M2. The sample rate was set at 2 kHz, and the vertical resolution was 24 bits. Before the positioning, each electrode site was abraded with a scrubbing gel (Nu Prep, Spes Medica, S.r.l, Genova, Italy); then, a conductive cream (Neurgel, Spes Medica, S.r.l., Genova, Italy) was used to balance and decrease the contact impedance below 10 kΩ [91]. The recording was controlled by the NeoRec software v. 1.5.13 (Medical Computer Systems, Ltd.). 

The SC and PPG signals were acquired using, respectively, the SA9308M and SA9309M (Thought Technology, Ltd., Montreal, QC, Canada) sensors connected to the FlexComp device (Thought Technology, Ltd., Montreal, QC, Canada). The 2 Ag/AgCl electrodes of the SA9308M were placed on the index and middle fingers of the non-dominant hand, while the SA9309M on the ring finger of the same hand. The SC was acquired using the constant voltage (0.5 V) mode [92]. Luminance variations were recorded by means of a T7670 sensor (Thought Technology, Ltd., Montreal, QC, Canada) attached to the top-right corner of the presentation screen. The sample rate was set at 256 Hz, and the vertical resolution was 14 bits. The recording was controlled by BioGraph Infiniti software v. 5.1.2 (Thought Technology, Ltd., Montreal, QC, Canada). 

iMotions software v. 10.0 (iMotions, A/V) was used to deliver the stimuli (i.e., redirection to the e-commerce and SL platforms) and the surveys. To establish an offline synchronization between the recorded data and the stimulus timestamps, two starting signals were generated at the beginning of the experiment: a TTL pulse, which was transmitted to the digital inputs of the NVX via the EEG Synchronisation Box (ESB) [93] and a fast visual pattern, consisting of black and white (B-W) alternating stimuli, which was captured by the T7670 sensor. 

### 2.3. Experimental Procedure

After signing the informed consent, participants entered the experimental room, where they were seated in a comfortable chair in front of a 23.8″ PC monitor (FlexScan EV2451 by Eizo KK, Hakusan, Japan). Then, two laboratory technicians placed the sensors and checked the signal quality before starting the recordings. This phase lasted about 10 min. The experiment began with a 60 s eyes-closed baseline (EYC), followed by a 120 s eyes-open baseline (BSL) consisting of a white fixation dot on a black background. Then, a shopping task in both the SL and e-commerce (EC) environments was presented in random order: in both scenarios, participants had to purchase the same product (a pair of sunglasses) from the same brand (Bondi). At the end of each task, a self-report questionnaire on the perceived quality of the experience—ease of use (PEOU), enjoyment (PE), informativeness (PI), flow, and cognitive effort (CES) was administered. These measures are further described in the Section 2.5. In order to avoid any effects resulting from the systematic exposure of the two environments in the same chronological sequence [94], the order of exploration of SL and EC was randomized among the participants [95]. To ensure a fair comparison with SL, PoiBo™ (https://poibo.it/ (accessed on 20 March 2024)), an e-commerce platform with a category-based layout offering the same products/brands as SL, was chosen for the experimental procedure.

#### Task Segmentation 

**Figure 1:**
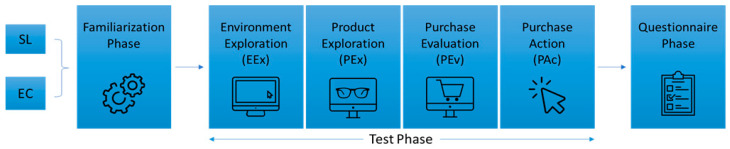
Task segmentation.

For both environments, the task consisted of a familiarization phase (not considered for analysis) and a test phase. Before the SL task, due to its complexity, participants followed a short tutorial on how to complete a purchase on the platform. The experience analysis was subdivided following the work of other authors who have tried to disentangle the complexity of the shopping experience [60,96]. Thus, the experience in both SL and EC was divided into four equal, specific phases (Figure 1):

- Environment Exploration (EEx): in this step, the participants freely explored both environments (EC by scrolling, and the world of SL with their avatar);
- Product Exploration (PEx): this phase regards participants’ interaction with the product they would later purchase. For SL, PEx was established at the moment they interacted with the product tab or tried it on; for EC, it was the moment when they were on the product page;
- Purchase Evaluation (PEv): for both SL and EC, this phase embedded 8 s before the actual purchase action. This was based on the fact that a purchase decision can be determined as early as 8 s before the actual purchase action [96]. The PEv phase did not overlap with the PEx phase;
- Purchase Action (PAc): this final phase refers to the exact moment of purchase, from the moment the person started to move the mouse to the purchase button on SL (“Add to Marketplace”) or EC (“Add to Cart”).

### 2.4. Data Processing

EEG, SC, and PPG signals were processed using MATLAB (The Mathworks, Inc., Natick, MA, USA), according to a previously adopted pipeline (see [66,97,98]).

The EEG was re-referenced to the linked earlobes, re-sampled to 512 Hz, and filtered using band-pass (0.1–30 Hz) and notch (50 and 100 Hz) filters. Then, non-stationary artifacts, such as movements and abrupt external noise, were corrected using artifact subspace reconstruction [99]. Stereotypical artifacts, such as eye blinking and muscular noise, were corrected using independent component analysis through the SOBI algorithm [100] and the ICLabel classifier [101]. Finally, a re-reference to the current source density was applied to increase the spatial resolution at the sensor level [102]. All the processing functions used were part of the EEGLab toolbox [103]. The cleaned EEG was aligned to the starting TTL pulse and epoched according to the onset and duration of the stimuli. For each subject, the individual alpha frequency (IAF), defined as the center of gravity of the power spectral density (PSD) within the extended alpha range (7.5–12.5 Hz) [104], was computed. In the IAF calculation, the mean PSD averaged across all the occipital channels was considered. The PSDs were computed on the EYC data according to Welch’s method with a 1 s long Hamming window and 50% overlapping. The IAF defined the 5 canonical EEG bands as δ = [0; IAF − 6], θ = [IAF − 6; IAF − 6], α = [IAF − 2; IAF + 2], β = [IAF + 2; IAF + 16], and γ = [IAF + 16; IAF + 25] [105]. 

The SC was band-pass filtered (0.001–0.35 Hz) and down-sampled to 32 Hz. Then, artifactual points were identified as those exceeding three thresholds (minimum amplitude of 0.05 µS, maximum amplitude of 60 µS, rate of change of ±8 µS/s) and replaced by a linear interpolation [106]. Finally, the skin conductance level (SCL) was computed using the cvxEDA algorithm [107]. 

The PPG was low-pass filtered (5 Hz) and down-sampled to 32 Hz. Then, peaks were identified using the Pan–Tompkins algorithm [108], and the instant Heart Rate (HR) was computed from the inverse of the peak-to-peak distance. Finally, the HR signal was linearly interpolated and filtered with a 2 s long moving average filter. 

The SCL and HR signals were aligned to the starting B-W pattern and epoched according to the onset and duration of the stimuli. From the EEG signal, several indicesbased on spectral instant powers within specific bands and electrodes were computed. Their calculation was based on the short-time Fourier transform with a 1 s long Hamming window and 50% overlapping as it has shown better performances compared to the filtering approach [109]. 

The beta over alpha plus theta ratio (BATR), or engagement index, was obtained by the ratio between the β and the sum of α and θ powers averaged across the entire set of electrodes. It has been proposed as a measure of cognitive engagement related to visual attention [110,111]. 

The workload index (WL), or cognitive load, was obtained by the ratio between the θ and α powers averaged across the frontal and parietal electrodes, respectively. It has been proposed as a measure of mental workload associated with information processing and task execution [112,113,114].

The memorization index (MI) was obtained by averaging the θ powers on the prefrontal electrodes. It has been proposed as a measure of mnemonic retention for the observed stimulus: higher values have been associated with increased activation of memorization processes [115,116,117,118]. 

The emotional index (EI), obtained by combining SCL and HR into a unidimensional signal, indicates the strength of the emotional response: higher values correspond to more positive emotions and lower values to more negative ones [119,120].

BATR, WL, and MI had a temporal resolution of 0.5 s, while that of EI was 1/32 = 31 ms.

To reduce physiological inter-subject differences and to make the individual data comparable, all the indices were z-scored according to the mean and standard deviation computed in the BSL stimulus. Then, they were temporally averaged to obtain a condensed stimulus-related index [98].

For the sake of clarity, Table 1 describes the neurophysiological signals and indices used in the research.

### 2.5. Self-Report Questionnaire

The self-report questionnaire administered at the end of each shopping task (SL and EC) aimed to assess the perceived cognitive and emotional quality of the experience. Our methodology heavily relied on the Technology Acceptance Model, TAM [77], one of the most widely used models to study people’s acceptance of new technologies [121], such as the metaverse. 

TAM demonstrates strong reliability, convergent validity, and discriminant validity in evaluating perceived usefulness and future usage intentions of technological systems such as educational systems [122] and metaverse environments [123,124]. The relationship between TAM variables has been validated in multiple studies using statistical methods such as Pearson’s correlation coefficients and multiple linear regression. Additionally, the high Cronbach’s α values in TAM studies [123,124] indicate good internal consistency, further supporting its reliability.Finally, integrating TAM with neurophysiological data can lead to more comprehensive results that provide a deeper understanding of the phenomenon under investigation.

TAM comprises several dimensions, each contributing to the assessment of user acceptance of technologies. Specifically, we used the following dimensions: 

Perceived Ease Of Use (PEOU) refers to the extent to which users expect the target system to be free of effort [77,125]. It measures how intuitive and straightforward the system is perceived to be [126,127]. PEOU reduces the cost of learning and searching for information [128], enables greater use of the online environment [129], and is often considered a key construct for assessing user acceptance of online environments [127]. It has also been found to indirectly influence online purchase intention [128], predict online repurchase intention [130], and have a positive relationship with perceived website usefulness and user satisfaction [127]. This dimension was derived from Rese et al. work [131], which updated and adapted the scale originally created by Davis and colleagues [132]. The scale consists of 3 items (e.g., “I think the platform is easy to use”). Answers can be given on a 6-point Likert scale from 1 = “not at all” to 6 = “very much”. 

Perceived Enjoyment (PE) can be defined as “the extent to which the activity of using a computer is perceived to be enjoyable in its own right, apart from any anticipated performance consequences” ([132], p. 1113). It assesses the pleasure derived from using the system and seems to have a positive relation with attitudes towards online advertising and intentions to purchase advertised products [78,133]. In virtual environments, it is influenced by PEOU [134] and acts as a determinant of technology acceptance [134] in AR/VR environments [135,136]. This subscale originates from Rese et al. [131], who revised and modified the scale initially formulated by Davis and his team in 1992 [132]. Comprising three items (e.g., “Navigation on this platform is very fun”), responses were solicited on a 6-point Likert scale from 1 = “not at all” to 6 = “very much”. 

Perceived Informativeness (PI) is the user’s perception of the quantity and quality of information the system provides, measuring whether the system offers useful and comprehensive information [78]. It is a source of decision control that supports the completion of the purchase goal, ultimately giving consumers confidence through relevant product information to increase clarity about the product and arrive at a satisfying choice [131]. While not originally part of Davis’s TAM, this dimension has been effectively associated with the key components influencing behavior toward emerging technologies [78]. Indeed, PI seems to have a positive effect on online purchase intention in AR [137,138], focusing on the functional aspect of augmentation and highlighting the utilitarian aspect of innovation [134]. This dimension was refined and adjusted by Rese et al. [131]. It comprises three items (e.g., “How satisfactory do you find the shopping experience?”). Responses were solicited using a 6-point Likert scale from 1 = “not at all” to 6 = “very much”. Furthermore, the constructs of flow and cognitive effort were also measured. 

Flow is a psychological state in which a person simultaneously feels cognitively efficient, motivated, and happy [139,140]. It is considered a valid metric to measure user experience in online and virtual worlds, and a determinant of user acceptance and intention to use virtual worlds [141]. In an e-commerce environment, flow has good antecedents in liking [78,142], informativeness [78], and PEOU [142] and has a direct impact on purchase intention [78,143]. Flow was measured with the Flow Short Scale [80], which consists of 10 items (e.g., “I am totally absorbed in what I am doing”) that can be answered on a 6-point Likert scale from 1 = “not at all” to 6 = “very much”. 

Cognitive effort (CES) refers to the degree of engagement with demanding cognitive tasks. Closely related to motivation, difficulty, attention, and cognitive control [144], it has been negatively related to enjoyment in online shopping environments and found to negatively affect customer satisfaction [79]. In the metaverse, the cognitive operating costs of XR-mediated experiences vary: AR tends to be more expensive, VR less so, and both are generally more expensive than real-world experiences [31]. Four items from Karasek and Theorell’s scale [81] were used to measure CES to obtain information regarding the difficulty of the task (e.g., “The task was mentally very fatiguing”). Answers can be given on a 6-point Likert scale from 1 = “not at all” to 6 = “very much”. 

The decision to use a 6-point answer scale for each questionnaire item was made to achieve greater precision in measurement and to discourage neutral responses by removing the middle option [145]. For each dimension, the final value for each condition (SL and EC) was obtained by averaging the single-item values within the scale. 

### 2.6. Statistical Analyses

Statistical analyses were all performed using JASP v. 0.18.3.0, an R-based statistical software package [146]. For each of the neurophysiological indices(BATR, WL, MI, and EI), we applied a 2-way repeated measures ANOVA with Environment (2 levels: SL and EC) and Phase (4 levels: EEx, PEx, PEv, and PAc) as factors. The sphericity assumption was tested in advance using Mauchly’s test, and in the case of violation, the Greenhouse–Geisser correction was applied. Post hoc t-tests were corrected for multiple comparisons using Holm’s method. For the self-report measures, we performed a 2-way repeated-measures ANOVA with the Scales (5 levels: PE, PI, PEOU, flow, and CES) and the Environment (2 levels: EC and SL) as factors. The sphericity assumption was tested in advance using Mauchly’s test, and in the case of violation, the Greenhouse–Geisser correction was applied. Post hoc t-tests were corrected for multiple comparisons using Holm’s method. Additionally, the internal validity of each measure was assessed by Cronbach’s α. 

For both EC and SL environments, Spearman’s correlations (ρ) between neurophysiological indices on the whole task and the scales were computed using Python (v. 3.12.3) and Statsmodel (v. 0.14.19) library. *p*-values were corrected for a total of [(9 × 9) − 9]/2 = 36 multiple comparisons using Holm’s method. Heatmaps were drawn using Python and the Matplotlib (v. 3.8) library.

## 3. Results

### 3.1. Neurophysiological Results

**Figure 2:**
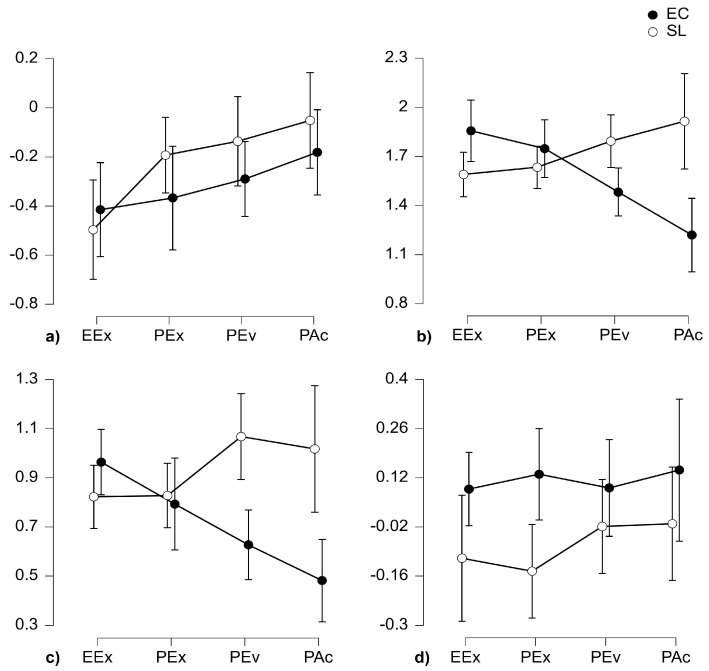
Descriptive plots with 95% CI error bars of BATR (**a**), WL (**b**), MI (**c**), and EI (**d**), split according to phase (EEx, PEx, PEv, PAc) and environment.

Descriptive statistics (means, M, and standard deviations, SD) and descriptive plots with 95% confidence interval (CI) bars are reported in, respectively, Table 2 and Figure 2.

BATR (Figure 2a) showed a significant main effect of Phase, F(2.107, 61.103) = 8.620, *p* < 0.001, η^2^ = 0.068, ϵ = 0.702. Post hoc comparison showed a significant difference between the first phase BATR_EEx_ and the last phase BATR_PAc_ (mean difference = −0.340, SE = 0.069, t(29) = −4.925, *p* < 0.001, d = −0.365). A nearly significant difference was also found between the second phase BATR_PEx_ and the last phase BATR_PAc_ (mean difference = −0.164, SE = 0.068, t(29) = −2.374, *p* = 0.059, d = −0.176). No significant differences were found for Environment or the interaction Environment × Phase. 

WL (Figure 2b) showed a significant Environment × Phase interaction, F(2.346, 60.996) = 16.012, *p* < 0.001, η^2^ = 0.150, ϵ = 0.782. Post hoc comparison showed a significant difference in the last phase between WLP_Ac_(SL) and WLP_Ac_(EC) (mean difference = 0.695, SE = 0.137, t (26) = 5.092, *p* <.001, d = 0.611). 

MI (Figure 2c) highlighted a significant Environment × Phase interaction, F(2.128, 61.712) = 9.702, *p* < 0.001, η^2^ = 0.068, ϵ = 0.709. Post hoc comparison showed a significant difference between MI_PEv_(SL) and MI_PEv_(EC) (mean difference = 0.441, SE = 0.120, t(29) = 3.688, *p* = 0.009, d = 0.550) and between MI_PAc_(SL) and MI_PAc_(EC) (mean difference = 0.536, SE = 0.120, t(29) = 4.482, *p* < 0.001, d = 0.668). 

EI (Figure 2d) showed a significant main effect for the Environment, F(1,31) = 4.171, *p* = 0.050, η^2^ = 0.053, highlighting lower values of the index in the SL condition in comparison to the EC condition (mean difference = −0.184, SE = 0.090, t(31) = −2.042, *p* = 0.050, d = −0.349). 

### 3.2. Self-Report Measures

**Figure 3:**
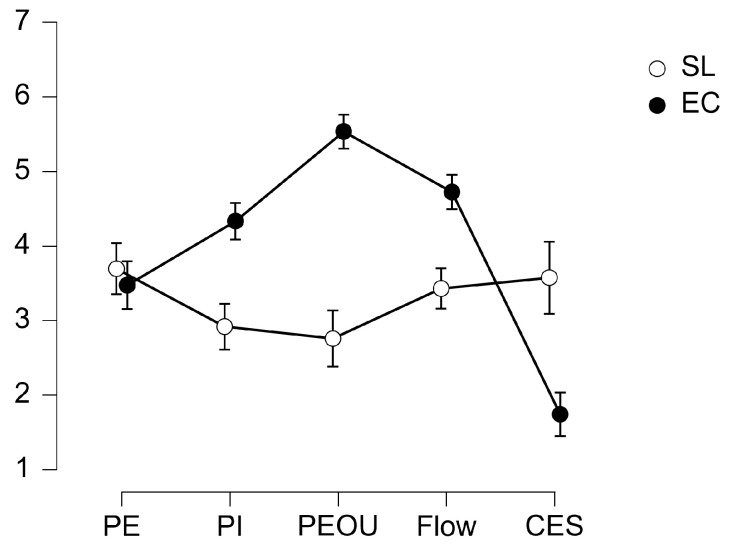
Descriptive plots with 95% CI error bars of the self-reports split according to dimension (PE, PI, PEOU, flow, CES) and environment (SL—white dot, EC—black dot).

Overall, the self-report measures for both the SL and EC environments showed Cronbach’s alpha (α) values ranging from 0.735 to 0.907, interpreted as “satisfactory” [147]. The α values did not significantly differ across the environments (Wilcoxon signed-rank test: W = 14.00, z = 1.753, *p* = 0.125). Descriptive statistics (mean, or M; standard deviation, or SD; and Cronbach’s α) and descriptive plots with 95% confidence interval (CI) error bars are reported in, respectively, Table 3 and Figure 3.

Significant main effects of both Environment (F(1,32) = 41.798, *p* < 0.001, η^2^ = 0.068) and Dimension (F(2.376, 76.032) = 23.387, *p* < 0.001, η^2^ = 0.164, ϵ = 0.595), as well as a significant Dimension × Environment interaction (F(4, 32) = 87.839, *p* < 0.001, η = 0.361), were found. Post hoc tests on the interaction Dimension × Environment highlighted significantly lower values for both PI_SL_ in comparison to PI_EC_ (mean difference = −1.141, SE = 0.199, t(32) = −7.106, *p* < 0.001, d = −1.377) and PEOU_SL_ in comparison with PEOU_EC_ (mean difference = −2.778, SE = 0.199, t(32) = −13.959, *p* < 0.001, d = −2.704). No significant result was found in the comparison between PI_SL_ and PI_EC_. Post hoc tests also showed significantly lower values for Flow_SL_ in comparison to Flow_EC_ (mean difference = −1.294, SE = 0.199, t(32) = −6.502, *p* < 0.001, d = −1.260) and significantly higher value for CES_SL_ compared to CES_EC_ (mean difference = 1.833, SE = 0.199, t(32) = 9.123, *p* < 0.001, d = 1.785).

### 3.3. Correlations 

**Figure 4:**
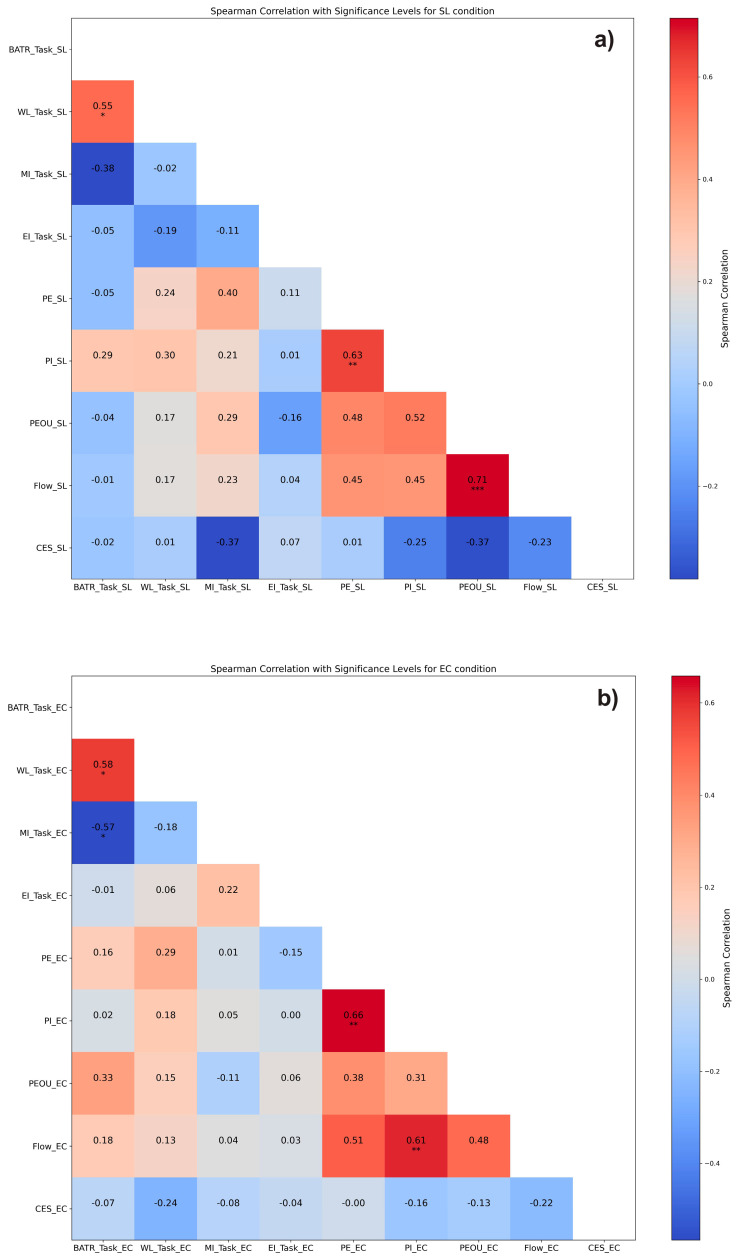
Heatmaps of Spearman’s correlations for both SL (**a**) and EC (**b**) environments. Significance levels are marked as follows: * *p* < 0.05, ** *p* < 0.01, *** *p* < 0.001.

In the SL condition (Figure 4a), a correlation was found between the neurophysiological index WL_TaskSL_ and BATR_TaskSL_ (ρ = 0.55, *p* < 0.05). Among the self-report dimensions correlations were found between PE_SL_ and PI_SL_ (ρ = 0.63, *p* < 0.01) as well as between Flow_SL_ and PEOU_SL_ (ρ = 0.71, *p* < 0.001). 

In the EC condition, the positive correlation between WL_TaskEC_ and BATR_TaskEC_ was confirmed (ρ = 0.58, *p* < 0.05). Additionally, BATR_TaskEC_ showed a negative correlation with MI_TaskEC_ (ρ = −0.57, *p* < 0.05). Among the self-report dimensions, correlations were found between PI_EC_ and PE_EC_ (ρ = 0.66, *p* < 0.01) and Flow_EC_ and PI_EC_ (ρ = 0.61, *p* < 0.01).

## 4. Discussion

This study aimed to disentangle the cognitive and emotional aspects of the shopping experience in two different digital retail platforms: an e-commerce (EC) environment and a metaverse environment, Second Life (SL). In particular, we tested whether a still-active and economically wealthy first-generation platform such as SL could perform equally or even better than traditional EC in the shopping experience. Performance was assessed by combining self-report and consumer neuroscience data. The shopping experience was split into four phases to gather information on environmental exploration (EEx), product exploration (PEx), product evaluation (PEv), and purchase action (PAc). Neurophysiological measures of engagement (BATR), workload (WL), memorization (MI), and emotion (EI) were collected, alongside self-report measures such as perceived ease of use (PEOU), perceived enjoyment (PE), and perceived informativeness (PI) from the Technology Acceptance Model (TAM), as well as flow and cognitive effort (CES). 

The first research question (RQ1) asked whether cognitive and affective states highlighted a more favorable pattern in the SL compared to the EC shopping experience. Neurophysiological findings revealed a complex interconnection between cognitive and emotional processes, highlighting the differences between the two experiences. Despite negative values for both SL and EC, cognitive engagement (BATR) showed steady and continuous growth over time, regardless of the environment (Figure 2a). Significant differences between the first (EEx) and last (PAc) experience phases confirm that engagement tends to increase as the interactivity with the environment increases [148]. Since cognitive engagement is a task-related cognitive demand to process visual and environmental stimuli [110,111], its higher levels in the PAc phase may suggest, independently of the environment, a higher level of cognitive resources needed to achieve the task goal [114,148]. 

In contrast from BATR, workload (WL) was environment-sensitive in the last phase (PAc), showing significantly higher values in SL. WL is defined as the cognitive cost of performing a task that can result in subjective discomfort and impaired performance [112,113,149,150]. These results suggest that although SL did not lead to a constant experience of cognitive overload, the purchase action may have been particularly demanding. Although a moderate workload is necessary to maintain motivation [151], it is often associated with a decline in performance [114] and increased task difficulty [152,153,154]. Moreover, recent studies have linked it to decreased happiness during the shopping experience [155], a negative impact on retail image [156], poor decision quality [157], and less immersive experiences in online retail [158]. The positive and significant correlation found in both EC and SL conditions between BATR and WL (Figure 4b) can be explained by the common nature of the two indices [104], as the mental workload is part of the cognitive resources needed to complete a task [159,160]. 

Memorization (MI) showed higher significant results in SL for both the purchase evaluation (PEv) and the final purchase action phase (PAc), similar to WL. Despite no significant correlation being found between MI and WL, the two indices share a common θ wave origin [114]. Indeed, cognitive workload influences the effectiveness of new information retention [114], which is described as the ability to manipulate and retain new information for a short period [161]. Since the last phases of the SL experience resulted in a highly cognitively demanding task, consumers may activate memorization processes to elaborate and retain new information, confirming the cognitive load as a possible condition to increase working memory load which boosts data retention [162]. 

Finally, EI showed positive values for both SL and EC conditions, but a significantly higher overall level of positive emotional involvement in the EC condition. In e-commerce, both traditional methods [163] and consumer neuroscience techniques [164] showed a positive relationship between positive emotional involvement and purchase intention. Higher levels of arousal and positive affective states are related to the willingness to seek new elements and explore novel assets in a virtual environment, including a web-based desktop platform [32], enhancing the immersive experience [165], boosting consumers’ creativity [166] and their sense of presence [167,168], and promoting purchase intention [169]. The lower emotional involvement in SL may be related to the cognitively demanding features of the environment, confirming the interconnection between cognitive and affective states [170,171,172]. Indeed, recent evidence suggests that more cognitively demanding tasks can inhibit the cortical and subcortical brain structures involved in emotional responses [173], indicating that higher levels of cognitive effort may negatively affect emotional states [174].

These complex results lead us to consider the SL experience as essentially more cognitively demanding than EC, especially for the final purchase action. This higher cognitive resource allocation would also be explained by the novelty of the SL shopping experience compared to traditional EC. Novelty in virtual/digital environments leads to heightened interest [175] and greater use of cognitive resources to seek new and meaningful information [176]. However, this possible explanation is not supported by the EI results, as novelty is often associated with increases in arousal and emotional intensity [176]. Instead, the highlighted cognitive and emotional pattern would confirm workload as a possible environmental over-engaging outcome [160] and the experience in this metaverse platform as highly cognitively demanding [31,45]. 

Therefore, addressing RQ1, it cannot be concluded that SL performs better than traditional e-commerce in terms of cognitive and emotional neurophysiological parameters. This consideration seems to be confirmed by the self-report results. 

RQ2 asked whether the SL experience was perceived as better than a traditional EC by asking participants to evaluate the experience through several dimensions. Perceived ease of use (PEOU) and perceived informativeness (PI) showed significantly higher values in the EC condition, leading users to consider the e-commerce experience more effortless [77], intuitive [126,127], and comprehensive [78] than SL. Indeed, according to recent research, both PEOU and PI positively influence online purchase intention [137,138] and the willingness to further engage with the digital environment [134]. In particular, PEOU is recognized as an antecedent of flow [142], which also resulted significantly higher in the EC. This would confirm a more cognitively demanding experience in SL, as flow is connected to perceived cognitive fluency and positive emotional experience [139,140], and cognitive load has been related to lower levels of flow in virtual environments [177]. Finally, the SL condition led to significantly higher values of perceived cognitive effort (CES), confirming the interpretation of neurophysiological results. CES reduces enjoyment in the online shopping environment as well as customer satisfaction [79]. However, in this study, no significant difference was found in perceived enjoyment (PE) between SL and EC.

Therefore, addressing RQ2, it cannot be concluded that the SL digital shopping experience was rationally perceived as better than traditional e-commerce. 

Combining both neurophysiological and self-report results, the SL condition confirms that environmental features in metaverse platforms may lead to higher cognitive demand [45,178], especially compared to traditional e-commerce [179]. However, as positive emotional experience is one of the key elements in virtual and digital online shopping experiences [32,169], lower results in SL indicate non-optimized overall immersion [180]. The environmental features and navigation issues, as well as low-resolution graphics and immersive experience [181], which have been addressed as part of SL’s problems [35], may have led to excessive cognitive demand [45], especially during the purchase action, resulting in a poor emotional experience [180] and lack of flow [177,182,183]. 

In addition to these findings, an important consideration is related to the nature of the sample, consisting of both Generation Z (Gen Z) and Millennials or Generation Y [82], two of the most active generations in the metaverse in the present and future perspectives [83]. These generations exploit the dramatic immersive experience of the new platform to bond virtuality and reality, merging online and offline selves [184]. Indeed, the online shopping experience of Gen Z is highly technology-sensitive [185] and driven by high expectations for electronic processes and e-solutions [186], as well as fun, enjoyment, and entertainment [187]. Therefore, spatial and visual constraints related to the PC-based metaverse platform [184] may result in a poor overall experience [188], especially for the newest generation. Considering the growing number of users in SL [34], its expansion as a virtual marketplace [33], and recent Gen Z-targeted sales activities and marketing campaigns [48], it is questionable whether these investments, by both companies and consumers, are supported by an effective elicitation of positive affective states and cognitive flow, crucial to engage customers in a functional online shopping experience [189,190].

### 4.1. Managerial Implications

This study’s findings offer valuable insights into the inclusion criteria of metaverse platforms in the digital shopping experience, with potential economic and managerial implications. They also confirm the added value of a holistic approach to this framework with the inclusion of consumer neuroscience. 

Although SL is considered a prolific digital shopping environment [36,37] with a growing number of active users [34] and a fruitful marketplace [33] with marketing activities targeting the youngest generation [48], our results suggest that companies should be cautious about investing in SL virtual retail. A tailored and in-depth analysis should always be addressed as environmental and functional features can influence cognitive and emotional states [32,169,180], modify user experience [188,191,192,193], and alter purchase intention [194], affecting the overall brand evaluation [195]. These considerations are crucial, especially for an early-stage field such as metaverse retailing [6], with no well-defined boundaries [184] but a growing investment forecast [5]. The match between consumers’ expectations and the effective performance of a digital shopping platform is a key point to building a valuable experience [22], preventing consumers from choosing traditional retail channels [6,196] and from abandoning the platform early on [197,198]. This consideration appears especially true for future investments, which will affect the newest generations, major adopters of the metaverse platforms for the digital shopping experience [83] which are mainly driven by high technological and electronic expectations [186] and emotionally arousing online shopping experiences [187]. 

The discussed need for a holistic analysis leads us to the last main implication of the study. As highlighted by scholars [51,52], consumer neuroscience and neuromarketing may help companies and scholars to deeply understand cognitive and emotional processes in response to virtual and digital environments [53]. Using EEG-related measures and autonomic parameters (e.g., SC and PPG), it is possible to gather information on perceptual, cognitive, and emotional processes [56,57], as well as behavioral changes [32], helping companies and scientific research to advance their knowledge on this topic and optimizing consumer experience in virtual retail. Combining traditional and neuroscience techniques, it is possible to gain a broader knowledge of consumer experiences in the digital shopping experience [199], addressing a wide range of challenges and questions such as the inclusion criteria for platforms in the metaverse framework and the effective user experience in the online digital platforms [6]. 

### 4.2. Limits and Future Directions

This work represents a pilot investigation into the metaverse and digital shopping platforms using consumer neuroscience techniques, however, it is not without limitations. The first limitation is the sample size, which is considered appropriate for initial exploration and neurophysiological investigation but may affect the generalization of the results to a broader population. For this reason, the authors call for further investigation with a larger sample size. The second limitation is the number of e-commerce and SL virtual retail stores that were tested, as well as the purchased products. This pilot study tested only one SL retail environment and one e-commerce environment, with a common and specific product to purchase. Thus, our results must be interpreted within these circumstances, although they offer a valuable starting point. Further research can replicate this study including other SL retail and e-commerce environments as well as other products. A third limitation is the target, as the whole sample included both Generation Z and Millennials. The reported results are related to a specific age range, and it is not possible to conclude that the data would be different from those of other generational targets. Although this is a clear limitation, the choice was debated in the discussion and in the subsection on managerial implications. A fourth consideration is the cultural and socio-demographic background of the sample, since the test subjects are all Italian. For this reason, it is not possible to extend the results to different cultures. Future possible research may rise from the discussed limitations with a wide range of opportunities. Multiple metaverse platforms can be tested with different environmental features as well as different types of e-commerce by enrolling a larger and wider sample, taking into account different generations and cultural backgrounds.

## 5. Conclusions

This study aimed to investigate the difference in the shopping experience between a traditional e-commerce and a first-generation metaverse platform such as Second Life (SL). Despite previous authors highlighting a more engaging, immersive, and effortless shopping experience in SL in comparison with traditional e-commerce, our neurophysiological and self-report measures cannot confirm those findings. SL condition showed higher levels of cognitive engagement, but more effortful and cognitively demanding requests during the final purchase action, with higher levels of workload and memorization processes. Additionally, SL resulted in a less overall emotionally engaging experience. Both cognitive overload and emotional experience are important features to evaluate the effectiveness of a digital shopping experience, with important consequences for purchase intention and overall brand evaluation. Self-report measures also confirmed neurophysiological findings, showing that SL was perceived as more cognitively effortful, eliciting a lower level of flow, perceived ease of use, and perceived informativeness. These results lead to several implications, as metaverse retailing is a relatively new phenomenon in the digital field, and its challenges are still to be addressed. Since SL is still a flourishing and active marketplace with a growing number of users, companies and managers should always address a tailored holistic analysis before investing in the platform or creating an SL virtual retail. Our considerations are boosted by the chosen sample—Gen Z and Millennials—who are major actors in the metaverse framework and whose online shopping experience is driven by high technological expectations and hedonistic drivers. This study does not aim to discourage investment in SL, which remains one of the most enjoyable first-generation platforms, but suggests a more in-depth analysis to create and optimize virtual stores, preventing possibly rapid abandonments and consumer switching to traditional retail channels. Finally, the systematic usage of a mixed methodology combining both neuroscientific and traditional techniques may lead to an increase in the analytic power of the platforms and the metaverse framework. This would result in a model that companies and managers can use to optimize their investments and gather new information on brain response to the digital environment, contributing to enriching the knowledge of neuroscience and behavioral dynamics in this field. As digital platforms continue to evolve, addressing these issues and related problems will be critical to create an environment where consumers can conveniently and intuitively shop online, allowing both digital environments and the metaverse to reach their full potential as commerce platforms.

## Figures and Tables

**Table 1:** Neurophysiological signals and indices employed for the analysis.

Signal	Index	Analysis	Reference
EEG (Electroencephalogram)	BATR (Cognitive Engagement)	Measures the cognitive demand to process visual and environmental stimuli.	[111,112]
	WL (Workload)	Measures the cognitive cost of performing a task.	[113,114,115]
	MI (Memorization)	Measures the potential activation of memorization processes.	[107,108,109,110]
SC (Skin Conductance)	EI (Emotional Index)	Combines both SCL measured via SC sensors and HR measured via PPG. It indicates the emotional strength and valence of the experience.	[116,117]
PPG (Photoplethysmogram)			

**Table 2:** Descriptive statistics (mean—M, standard deviation—SD) of BATR, WL, MI, and EI split according to phase (EEx, PEx, PEv, PAc) and environment (SL, EC).

Environment	Phase	BATR		WL		MI		WI	
		M	SD	M	SD	M	SD	M	SD
SL	EEx	−0.497	1.056	1.589	1.136	0.823	0.758	−0.109	0.417
	PEx	−0.192	0.924	1.633	1.032	0.827	0.577	−0.146	0.430
	PEv	−0.136	0.946	1.793	1.207	1.068	0.816	−0.019	0.500
	PAc	−0.051	0.980	1.915	1.340	1.018	1.017	−0.011	0.567
EC	EEx	−0.415	0.864	1.856	1.015	0.964	0.781	0.088	0.452
	PEx	−0.367	0.917	1.748	1.007	0.793	0.805	0.130	0.561
	PEv	−0.290	0.792	1.482	1.117	0.627	0.729	0.091	0.584
	PAc	−0.181	0.949	1.219	1.209	0.482	0.864	0.142	0.661

**Table 3:** Descriptive statistics (mean—M, standard deviation—SD, and Cronbach’s α) of the self-reports split according to dimension (PE, PI, PEOU, flow, CES) and environment (SL, EC).

Environment	Dimension	Mean	SD	Cronbach’s α
SL	PE	3.697	1.234	0.874
	PI	2.919	1.202	0.839
	PEOU	2.758	1.265	0.907
	Flow	3.430	0.999	0.902
	CES	3.576	1.157	0.901
EC	PE	3.457	1.187	0.905
	PI	4.333	0.946	0.795
	PEOU	5.535	0.623	0.843
	Flow	4.724	0.669	0.760
	CES	1.742	0.683	0.735

## Data Availability

Data are available on request from the authors.
